# Type 3 finger length pattern is associated with total knee replacements due to osteoarthritis but not with hip replacements or hand osteoarthritis in the elderly: The AGES-Reykjavik study

**DOI:** 10.1186/1471-2474-14-112

**Published:** 2013-03-26

**Authors:** Kristin Sigurjonsdottir, Thorir M Bjorgulfsson, Thor Aspelund, Gudny Eiriksdottir, Sigurdur Sigurdsson, Thorvaldur Ingvarsson, Tamara B Harris, Lenore Launer, Vilmundur Gudnason, Helgi Jonsson

**Affiliations:** 1 University of Iceland, Reykjavik, Iceland; 2 Icelandic Heart Association, Kopavogur, Iceland; 3 Akureyri Central Hospital, Akureyri, Iceland; 4 National Institute on Aging, Bethesda, MD, USA; 5 Landspitalinn University Hospital ICELAND, Reykjavik, Iceland

**Keywords:** Osteoarthritis, Finger length ratio, Epidemiology

## Abstract

**Background:**

Recent case–control studies have shown an association between type 3 finger length pattern (longer ring finger than index finger) and knee osteoarthritis. This large cross-sectional study tests the hypothesis that the type 3 pattern is associated with total joint replacements due to osteoarthritis in a large population based study.

**Methods:**

Finger length ratios were assessed visually on 5170 hand photographs (2975 females, 2195 males, mean age 76). In this population-based multidisciplinary study of aging in Reykjavik, Iceland, the prevalence of osteoarthritis associated total knee replacements was 223(4.3%) and total hip replacements 316(6.1%). We then performed a binary logistic regression analysis for total knee replacements and total hip replacements, including finger length patterns, osteoarthritis at other sites and other variables with possible association to osteoarthritis such as age, BMI and bone mineral density of the spine.

**Results:**

The prevalence of the type 3 pattern was 50% (43% in females, 58% in males). The regression analysis revealed an odds ratio for total knee replacements of 1.65 (1.24-2.2) p = 0.0007, in the type 3 finger pattern group, similar in both genders. This association was independent of the associations we have previously reported between total knee replacements and BMI and the presence of hand osteoarthritis. No association was seen between finger length patterns and total hip replacements.

**Conclusion:**

Finger length patterns read from digital photographs in this large study confirm previous radiographic observations with significant associations between the type 3 pattern and total knee replacements but not total hip replacements in both genders in this elderly group.

## Background

Osteoarthritis (OA) is the most common form of arthritis. It is a major cause of disability in middle aged and older people. The etiology appears to be a combination of systemic and local factors. Several risk factors have been identified, such as obesity, higher age and joint injury but in recent years evidence is growing for the importance of systemic factors such as genetics, hormones and bone density [1].

The index to ring finger length ratio (2D:4D ratio) is a sexually dimorphic trait, men tend to have a longer ring finger than index finger, while in women the fingers are more equal in length [2]. There is evidence that low 2D:4D ratio is a reflection of prenatal androgen exposure and the ratio therefore functions as a surrogate marker for androgen exposure in utero [3,4].

The 2D:4D ratio has been associated with a wide variety of physiological and behavioral traits. Type 3 finger pattern (longer ring finger than index finger) has been shown to be associated with several masculine characteristics such as achievement in sports and athletics [5-8], sperm counts [2], family size [9] and stock handling ability [10]. Type 3 finger pattern has also been associated with various medical conditions such as autism [11], ADHD [12] and prostate cancer [13,14].

Two recent case–control studies have shown an association between type 3 finger pattern and knee osteoarthritis [15,16], but subsequently, this association could not be confirmed in a population based study [17].

This large cross-sectional study tests the hypothesis that the type 3 finger pattern is associated with total joint replacements (TJR) due to osteoarthritis in a large population based study.

## Methods

The AGES-Reykjavik Study is a population-based multidisciplinary study of aging in the elderly population of Reykjavik, Iceland. The study is approved by the Icelandic National Bioethics Committee, (VSN: 00–063) and the Data Protection Authority. The recruitment to the study was finalized in January 2006 and there were 5764 participants aged between 66 and 96. Informed consent was obtained from all participants. The main object of the study was to examine aging and risk factors for cardiovascular disease but being large and multidisciplinary, the study has enabled us to look at other health issues as well, in this case OA [18]. The BMI and bone mineral density data used in the analysis were part of the study protocol.

Among the extensive baseline data gathered on the study participants was information about whether they had undergone total replacements of knees and hips and whether or not they had hand osteoarthritis. Knee and hip joint replacements (TKR, THR) were recorded on the basis of computerized tomography (CT) scans. Hand osteoarthritis was ascertained from digital photographs of the hands [19]. A total of 5250 individuals had both CT scans and hand photographs for the assessment of hand OA. A total of 53 participants were excluded because their hand photographs could not be scored for hand OA. After excluding those with inflammatory arthritis and fractures as the reason for total joint replacements (TJR) using previously described methods [20] we had information about TKR and THR presumably due to OA in 2195 males and 2975 females, mean age 76 ± 6 years [20]. The prevalence of TKR was 223 (4.3%) and THR 316 (6.1%). The prevalence of hand OA was based on photograph readings with a grading of 0–4 as previously described [19]. Those with a grade of 4 were considered to have severe hand OA (n = 673, (13%)).

Finger length ratios were assessed visually on the 5170 hand photographs. Two readers (KS,TB) assessed the photographs separately and classified them (based on both hands) as type 1 (index finger longer than the ring finger), type 2 (index finger equal to the ring finger) or type 3 (index finger shorter than the ring finger). In most cases the hands had similar finger length patterns, but occasionally there was a difference between hands. In these cases, an aggregate assessment was made. Thus a participant with a clear type 3 pattern on one side but equal length (type 2) on the other would be rated as type 3. A third reader (HJ) assessed every photograph where the other two readers had differed and decided the appropriate score.

For comparison, similar visual readings were done with 371 hand radiographs (211 females, 160 males mean age 75.6 ± 4.8) from the same group. The same two readers that classified the photographs also classified the radiographs based on visual assessment. Exact (pixel) measures of the phalanges were also obtained from the radiographs. The length of the phalanges was determined by measuring from the midpoint of the base of the proximal phalanx to the midpoint of the tip of the distal phalanx.

### Statistics

Statistical analysis was done with the SPSS 19 software package. Average intraclass correlation coefficient (ICC) was used to determine agreement between the two readers after they had classified the photographs. Calculated ICC between the two initial observers was 0.6. The reproducibility of the final visual classification was examined by using a sample of 125 random photographs where final scores had been reached twice in a blinded fashion. ICC for these two sets of data was 0.88. Binary logistic regression was used for calculating odds-ratios.

## Results

**Figure 1:**
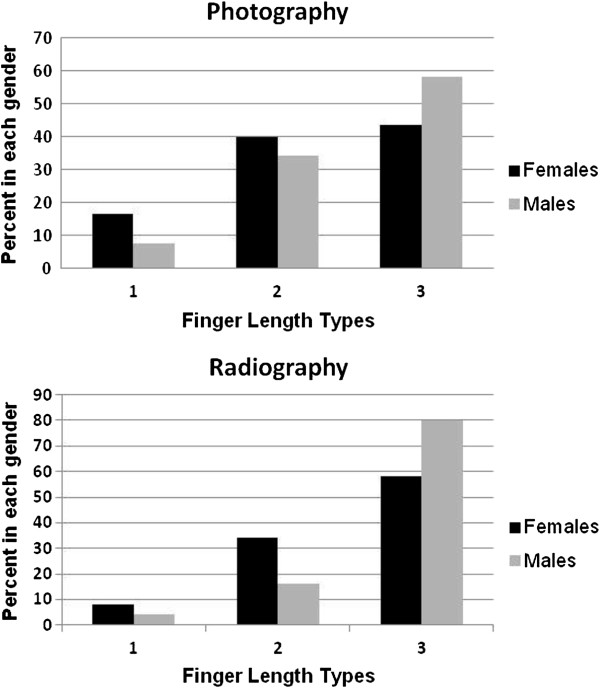
**Visual assessment of the photographs and the radiographs. **Visual assessment of the photographs tends to underestimate the prevalence of the type 3 finger length pattern compared to visual assessment of the radiographs.

Finger length ratio of the hand radiographs was assessed both visually and with exact measurement of the finger lengths. Visual assessments of the finger length ratio overestimated the pixel 2D:4D ratio which was on average 0.91 ± 0.02 and less than 1 in all cases (range 0.852-0.981). According to visual assessments of the radiographs, 64% had type 3 finger pattern (54% in females, 78% in males) (Figure 1).

Visual assessment of photographs had an even lower prevalence of the type 3 finger pattern 50% (43% in females, 58% in males). The Spearman correlation coefficient between the two methods was 0.65, and for the pixel ratio and the photographic reading was 0.56.

The population with TKR had a mean age of 76.9 years, 64% were female and mean BMI was 29.5. The population with TKR was older and had a higher BMI compared to the population without TKR. In the photographic assessment there were associations with age (higher prevalence of type 3 pattern) and height (lower prevalence).

The prevalence of type 3 finger length pattern was higher in the population with TKR compared to the population without TKR. In the population with TKR 61% had type 3 finger length pattern while in the population without TKR only 49% had type 3 finger length pattern. No such relationship was seen for THR or hand osteoarthritis.

In the radiographic assessment, the population with TKR was older and heavier than the population without TKR, similar to the photographic assessment. There was also a higher prevalence of type 3 finger pattern in the population with TKR compared to the population without TKR (Tables 1 and 2).

**Table 1:** Results for the photography population

**Photography population (n=5170)**							
		**Knee OA**		**Hip OA**		**Hand OA**	
		TKR(n=223)	No TKR (n=4947)	THR(n=316)	No THR(n=4854)	Severe(n=673) not severe(n=4497)	
Age, mean(SD) years		76.9(5.4)	76.4(5.5)	77.9(5.9)	76.4(5.5)	77.5(4.3)	75.4(4.8)
Percent female		64	57	57	65	73	55
BMI, mean (SD)		29.5(4.7)	26.9(4.4)	27.8(4.5)	27.0(4.4)	26.0(4.2)	27.6(4.3)
Finger pattern (visual photographic,%)							
	1	10	13	11	13	14	13
	2	30	38	36	38	33	38
	3	61	49	53	49	53	49

The prevalence of type 3 finger length pattern was higher in the population with TKR compared to the population without knee replacement. No such relationship was seen for THR or hand osteoarthritis.

**Table 2:** Results for the radiography population

**Radiography population (N=371)**						
	**Knee OA**		**Hip OA**		**Hand OA**	
	TKR(n=17)	No TKR (n=354)	THR(n=24)	No THR(n=347)		
Age, mean(SD) years	76.6(5.2)	75.6(4.8)	76.2(4.3)	75.5(4.8)	77.5(4.3)	75.4(4.8)
Percent female	72	56	58	57	73	55
BMI, mean(SD)	30.4(3.8)	27.2(4.3)	27.5(4.6)	27.4(4.3)	26.0(4.2)	27.6(4.3)
Finger pattern (visual,%)						
1	0	10	8	9	9	9
2	24	27	21	27	21	27
3	76	64	71	64	70	63
2D:4D finger ratio(SD)	0.902(0.022)	0.91(0.023)	0.904(0.023)	0.91(0.023)	0.902(0.022)	0.91(0.023)

We then performed a binary logistic regression analysis for TKR and THR, including finger length patterns, OA at other sites, age, gender, BMI and bone mineral density of the spine.

The regression analysis revealed an odds ratio for TKR of 1.65 (1.24-2.2) p = 0.0007, in the type 3 finger pattern group compared with the rest of the study group. It was similar in both genders, females had an OR of 1.62 (1.14-2.31) and males had an OR of 1.74 (1.05-2.86). There was no association between finger length patterns and severe hand OA (OR 1.19 (0.99-1.41), p = 0.053) nor THR (OR 1.14 (0.9-1.44), p = 0.3). In the much smaller radiography sample, unadjusted OR for TKR was 1.9 (0.6-5.8) in the type 3 finger group, not significant.

To account for the possibility that the presence of hand OA might be affecting finger length assessment we also did a separate analysis in the group with no evidence of hand OA on photographs (n = 1602). The OR for TKR in individuals with type 3 finger length in this group was similar (OR 1.88 (1.05-3.35), p = 0.034). The OR for THR was 1.01 (0.64-1.59, p = 0.96)

## Discussion

In this large population based study there is a clearly significant association in both genders between type 3 finger pattern and OA attributed TKR but not THR. The association is independent of other established risk factors for OA, such as age, gender, BMI and the presence of hand osteoarthritis. The results from this large population based study confirm previous reports regarding the positive association between type 3 finger pattern and knee OA. Zhang *et al.* (2008) [16] and Ferraro *et al.* (2009) [15] found the association to be stronger in women than in men but in our study the association is similar in both genders. Not all researchers have come to the same conclusion regarding this matter, Haugen *et al.* (2011) found an association between type 3 finger pattern and knee injury in men but they did not find significant associations with knee OA. However, among the 584 female participants in their study the 50 who had severe symptomatic knee OA had an adjusted odds-ratio of 1.8 (0.85–3.81) in the lowest 2D:4D ratio tertile, possibly indicating that the lack of significance may be a type 1 error due to lack of numbers [17].

The underlying mechanism accounting for the association between type 3 finger pattern and knee OA but not hip OA is unknown and we can only speculate on the subject. On the basis of current knowledge however, at least three possible mechanisms may be involved, the “post-traumatic”, the “morphometric” and the “hormonal”.

There is accumulating data that type 3 finger pattern is positively associated with athletic ability. It has been associated with achievement in many competitive and physically demanding sports [6,7,21]. Research has also indicated that individuals with type 3 finger pattern are more active and more likely to take risks [22]. Perhaps this makes them more prone to injury and repetitive joint microtrauma and that could make them more susceptible to OA. This is supported by the findings of Haugen *et al.* who found a higher reported knee injury rate in men with low 2D:4D ratios [17].

Type 3 finger pattern has been associated with a more masculine type of facial shape [23]. It is plausible that finger length ratios could be associated with size and shapes of other bones in the body. Knee osteoarthritis has been associated with knee height and possibly also to knee shape [24,25]. There is relatively little data available on this subject but morphometric characteristics of this type may well contribute to the findings in this study.

Last but not least we must consider a more direct hormonal relationship between knee OA and 2D:4D finger length ratio. Estrogen in particular has a complex and possibly protective relationship with the development of OA [26]. Current evidence does not suggest that finger length ratio is associated with adult hormone levels [27], but more subtle mechanisms may be operating. The lack of association between finger length ratio and hand osteoarthritis in this study however does not lend support to this theory.

Finally, there is the issue of genetics to be considered. Twin studies indicate that both finger length ratio and osteoarthritis are hereditary conditions [28,29]. These observed morphological changes in digits, knees and facial shape might by explained by a genetic variation that influences structural connective tissue morphology and function. Osteoarthritis seems to have a very complex genetic background with a multitude of candidate genes [30]. Finger length ratio on the other hand has been associated with a variant of the LIN28B gene [31], but to our knowledge there is no known sharing of associated genes between the two conditions.

Our study is limited by a number of factors. Instead of hand radiographs used in previous osteoarthritis studies, we used high quality photographs. We find that finger length patterns can be read from digital photographs but the method seems to underestimate the prevalence of type 3 finger pattern compared with radiographic readings. Age, height and metacarpal size may also contribute to a possible misclassification. The strength of the study lies in the large population based sample and the uniform OA cutoff using TKR as a marker for the group that has the most severe form of the disease.

This study confirms previous reports and we now have convincing evidence that type 3 finger pattern is associated with knee OA. We believe that a new risk factor for knee OA has been identified and should be a part of clinical assessment and risk stratification. However, further work is needed to improve our understanding of the mechanism responsible for this association.

## Conclusions

Finger length patterns read from digital photographs in this large study confirm previous radiographic observations with significant associations between the type 3 pattern and total knee replacements but not total hip replacements in both genders in this elderly group.

## Competing interests

The authors have declared no conflicts of interest.

## Authors’ contributions

KS, Main author. TMB, Data acquisition, Manuscript preparation. TA, Statistical analysis, Manuscript preparation. GE, Project management, Critical reading of manuscript. SS, Data acquisition, Critical reading of manuscript. TBH, Project management, Manuscript preparation. LL, Project management, Critical reading of manuscript. VG, Project management, Manuscript preparation. HJ, Data acquisition, Manuscript preparation. All authors read and approved the final manuscript.

## Pre-publication history

The pre-publication history for this paper can be accessed here:

http://www.biomedcentral.com/1471-2474/14/112/prepub
